# Comprehensive analysis of pedicle screw implantation in the C7 vertebra using computed tomography-based three-dimensional models

**DOI:** 10.1186/s12893-022-01548-5

**Published:** 2022-03-14

**Authors:** Huan Liu, Zhi-Yong Zhou, Jia-Xu Wei, Ming Zhang, Meng Bai, Ai-Bing Huang

**Affiliations:** 1 Department of Orthopedics, Hospital Affiliated 5 to Nantong University (Taizhou People’s Hospital), Taizhou, 225300 Jiangsu China; 2 Postgraduate School, Dalian Medical University, Dalian, 116000 Liaoning China

**Keywords:** Cervical pedicle screw, 3D, Entry point, Trajectory, Sagittal angle, Lateral angle

## Abstract

**Background:**

From a biomechanical point of view, pedicle screws (PS) are better than other kinds of screws for implantation in the seventh cervical vertebra (C7). However, the application of PS is limited because of the high risk of severe complications. It is essential to define the optimal entry point and trajectory. The aim of this study was to comprehensively analyze the starting point and trajectory for C7 PS insertion using three dimensional (3D) models.

**Methods:**

Overall, 60 subjects aged 18 to 67 years old were included. All CT images were used to construct 3D computer models of the C7 vertebrae. A new coordinate system was established for the next evaluation. The pedicle axis was calculated with respect to the entire pedicle; then, the ideal entry point, screw diameter and length, sagittal angle and lateral angle were assessed.

**Results:**

All the ideal entry points were located at the medial superior to lateral notch (LN), and the mean distance between the entry point and LN was 5.86 ± 1.67 mm in the horizontal direction and 3.47 ± 1.57 mm in the vertical direction. The mean distance between the entry point and the middle point of the inferior edge of the C6 articular process (MP) was 0.74 ± 1.83 mm in the horizontal direction. The mean sagittal angle of the pedicle axis was 90.42°, and the mean pedicle transverse angle was 30.70°. The average diameter and length of the PS were 6.51 ± 0.76 mm and 31.58 ± 4.40 mm, respectively.

**Conclusions:**

This study provided a novel method to calculate the ideal starting point and trajectory for C7 PS insertion. These measurements may be helpful for preoperative planning. It is recommended that 3D CT imaging is used preoperatively to carefully evaluate the anatomy of each individual.

## Introduction

The seventh cervical vertebra (C7) has unique morphological features; the lateral mass is less developed, and the pedicle dimensions are larger than those of other cervical vertebrae [1–3]. It is also a transitional vertebra between the cervical vertebra and the thoracic vertebra [2]. In multisegmental fixation of the cervical spine, C7 is usually the most caudal point and the site of the highest concentration of stress [4]. Therefore, pedicle screw (PS) fixation is the optimal choice.

Although a variety of techniques have been developed to improve PS placement accuracy, complications related to PS fixation remain an important concern [5–9]. Yukawa et al. [7] inserted 100 cervical PSs in C7 by a fluoroscopy-assisted pedicle axis view technique, and the postoperative CT scans showed that eight screws were sticking out of the pedicle. In another study reported by Nakashima et al. [9], the rate of PS misplacement in C7 was 12.9% (8 of 62), and neurovascular complications directly attributable to screw insertion were observed in some patients.

To optimally place a PS in C7, the ideal entry point and trajectory need to be identified. A few studies have proposed methods to calculate the ideal entry point and trajectory [2, 10–14]. Anatomical features, such as the lateral notch (LN), inferior articular process and center of the lateral mass were recommended as landmarks for the implantation of a PS in C7. However, consistent entry points and orientations have not been reported in the literature, and some study limitations, including a small number of specimens [10] and simple measurements performed with two-dimensional images, cannot be ignored [12]. To improve PS insertion accuracy, it is necessary to quantitatively identify the PS insertion point and the pedicle axis by using three-dimensional (3D) pedicle anatomical morphology.

In the present study, we aimed to identify the ideal entry point and trajectory for C7 PS implantation with the use of 3D computed tomography (CT) models. We hope to provide useful data for the preoperative planning of C7 PS fixation.

## Materials and methods

The study was approved by the Regional Ethics Board. We retrospectively reviewed 60 patients (31 males, 29 females) aged 18–67 years (mean 43 years) who underwent CT scans (120 kVp, 200 mA, Siemens Somatom Sensation 16, Germany) of the cervical vertebra at our hospital between June 2018 and January 2020; all patients underwent CT scans because trauma or disease in the cervical vertebra was suspected. The CT images had a slice thickness and pitch of 1 mm. Patients were excluded if they previously underwent spine surgery or had evidence of infectious, neoplastic, traumatic, or congenital spine anomalies or severe degenerative changes.

**Fig. 1:**
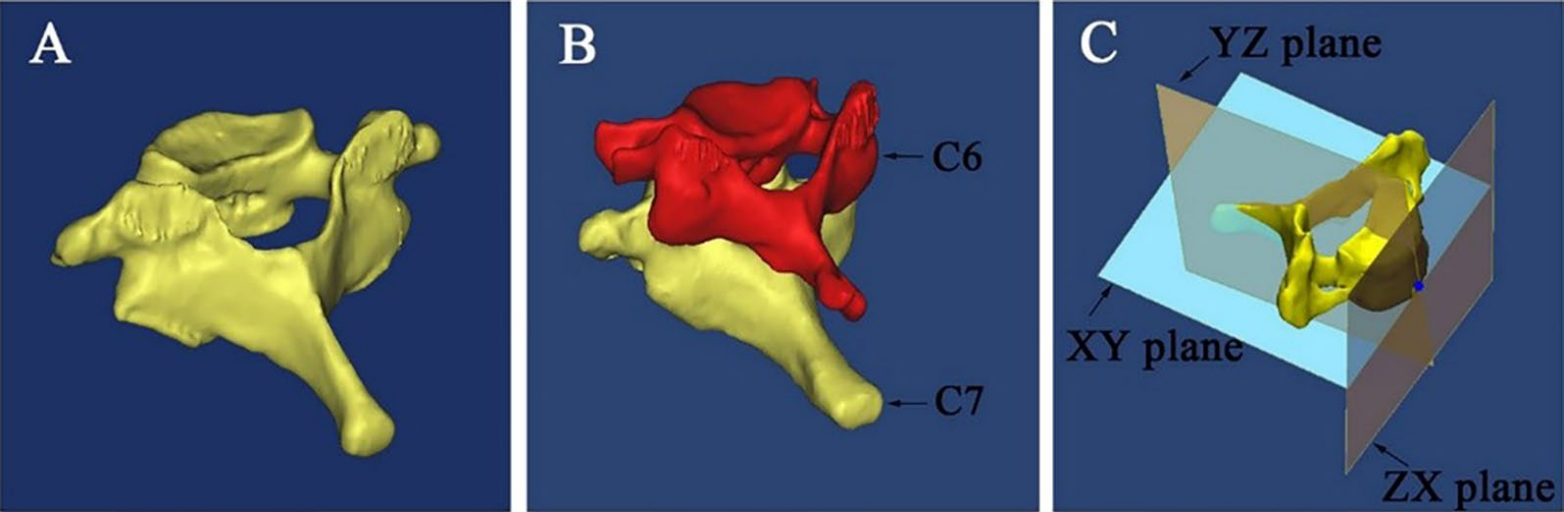
The construction of the 3D digital models from computed tomography images.** A** A 3D digital model of C7. **B** A 3D digital model of C7 with C6. **C** The model realigned with the new coordinate system

The CT data (DICOM format) were imported into Mimics software, version 17.0 (Materialise, Leuven, Belgium), to reconstruct the 3D digital models. To find and mark the middle point of the inferior edge of the C6 articular process (MP), we firstly reconstructed the 3D model of C7 with C6 and removed C6 for the next evaluation (Fig. 1).

To evaluate the C7 vertebrae anatomy with precision, the 3D digital models were imported into Geomagic Studio 2012 (Research Triangle Park, NC, USA) to establish a new coordinate system. The plane parallel to the superior endplate of the vertebral body was aligned with the XY plane, and the sagittal plane that bisected the vertebral body was aligned with the YZ plane. Then, the plane perpendicular to both of these planes was aligned with the ZX plane (Fig. 1).

**Fig. 2:**
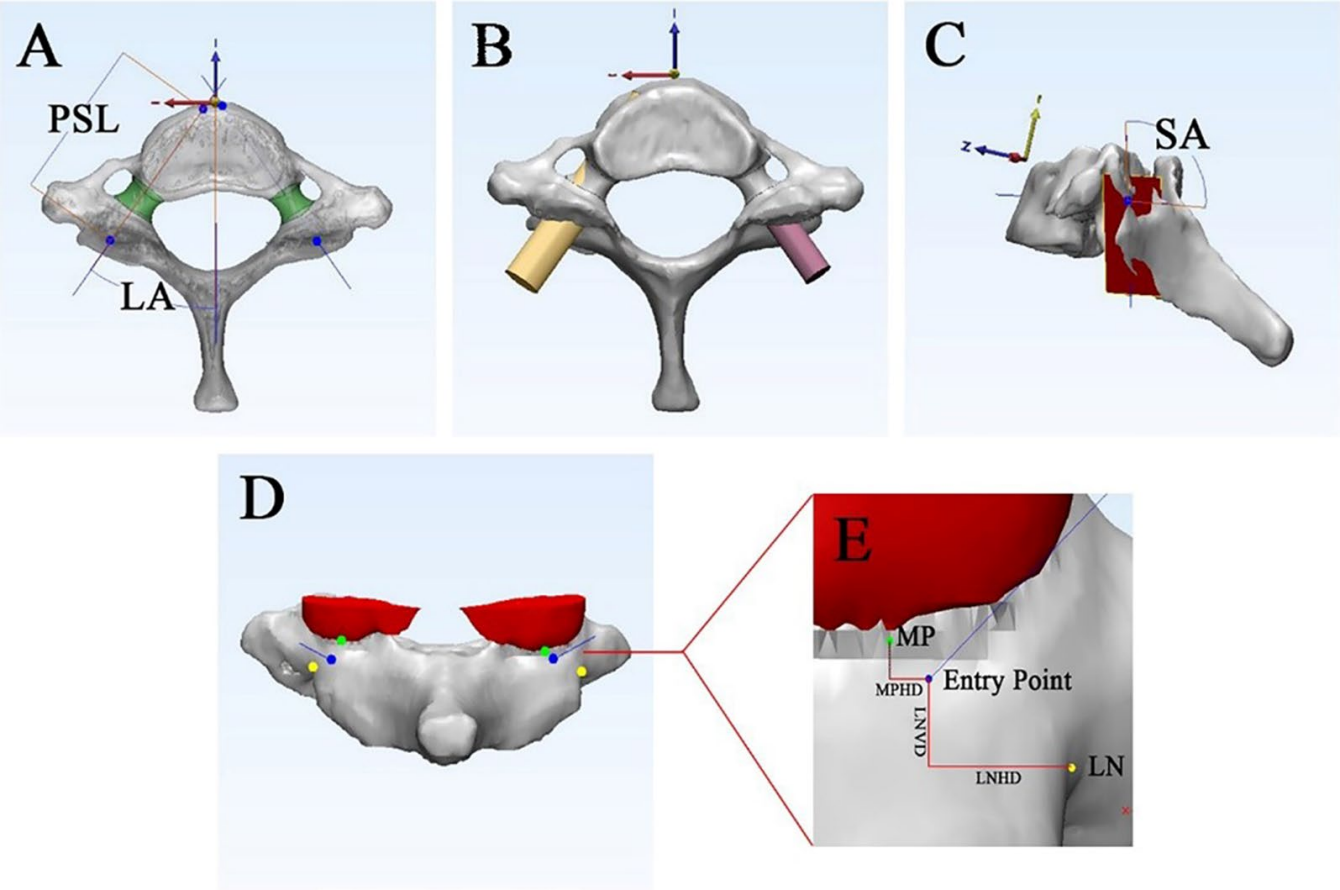
**A** The entire pedicle (green parts) was selected to determine the pedicle axis (blue lines). The pedicle lateral angle (LA) was defined as the angle between the axis and the YZ plane. The pedicle screw length (PSL) was defined as the distance between the entry point (intersection point of the trajectory and lateral mass surface) and intersection point (intersection point of the trajectory and anterior vertebral wall). **B** The pedicle screw diameter (PSD) was determined by the maximum diameter of the pedicle, which was automatically calculated by the software. The left side of pedicle, illustrating the pedicle was damaged by the simulation screw. **C** The pedicle sagittal angle (SA) was defined as the angle between the axis and the fitting plane of the lateral mass surface (red plane). **D** The locations of the entry point (blue point), LN (yellow point) and MP (green points) are illustrated. **E** The measurement of the lateral notch horizontal distance (LNHD), lateral notch vertical distance (LNVD) and middle point of the inferior edge of the C6 inferior articular process (MP) horizontal distance (MPHD)

The 3-matic medical 9.0 software system (Materialise, Leuven, Belgium) was used to determine the ideal trajectory and entry point and the diameter and length of the screws. First, the left pedicle of C7 was selected, and the pedicle axis was automatically calculated by the software. The point of intersection between the ideal trajectory and surface of the lateral mass was defined as the ideal entry point. Next, two bony landmarks (LN and MP) were identified and marked. We measured the linear distance between the entry point and LN and measured the horizontal distance between the entry point and MP. The LN horizontal distance and LN vertical distance were defined as positive when the entry point was located medial and superior to LN. The MP horizontal distance was defined as positive when the entry point was located lateral to the MP (Fig. 2). Because the sagittal location of the MP can be affected by the neck position, we did not measure the vertical distance between the entry point and MP. Finally, the PS diameter (PSD) was defined as the diameter of the largest cylinder the pedicle can accommodate. The PS length (PSL) was defined as the distance between the entry point and the point of intersection between the ideal trajectory and anterior vertebral surface (Fig. 2).

To determine the ideal screw trajectory, we also developed a new method of calculating the sagittal angle (SA), which was measured as the angle between the ideal trajectory and fitting plane of the lateral mass surface (FP). The angle between the ideal trajectory and the YZ plane was defined as the lateral angle (LA) (Fig. 2).

The intra- and interobserver agreement were assessed by the intraclass correlation coefficient (ICC). For intraobserver repeatability, all the measurements were repeated by the primary observer for 20 randomly selected cases. The primary observer was an attending surgeon with more than 15-year experience in spine surgery. For interobserver repeatability, all the measurements were repeated by an independent radiologist.

All statistical analyses were performed using SPSS software (version 21.0, SPSS Inc., Chicago, IL). The mean and the standard deviation were computed for all eight parameters. Two-factor analysis of variance was used to determine significant differences (p < 0.05) in each parameter between sexes and sides.

## Results

A total of 120 pedicles from the sixty 3D digital models of C7 were analyzed. Seven parameters were measured, including the LN horizontal distance, LN vertical distance, MP horizontal distance, SA, LA, PSD and PSL. The intra- and interobserver ICC values are summarized in Table 1. A high level of repeatability was demonstrated for the measurements of PSD, PSL, LA, SA, LN horizontal distance and LN vertical distance (the ICCs ranged from 0.76 to 0.96). A medium level of repeatability was demonstrated for the measurements of the MP horizontal distance (the ICCs ranged from 0.65 to 0.68).

**Table 1:** Coefficients of repeatability for interobserver and intraobserver measurement repeatability

	Interobserver	Intraobserver
LN horizontal distance (mm)	0.91	0.91
LN vertical distance (mm)	0.76	0.82
MP horizontal distance (mm)	0.65	0.68
SA (°)	0.85	0.88
LA (°)	0.89	0.85
PSD (mm)	0.94	0.96
PSL (mm)	0.81	0.85

*LN* lateral notch, *MP* the middle point of the inferior edge of the C6 articular process, *SA* sagittal angle, *LA* lateral angle, *PSD* pedicle screw diameter, *PSL* pedicle screw length

The parameter values and results of the statistical comparisons are shown in Table 2. There were no statistically significant differences between the different sides in the measured parameters, except for the LN vertical distance and SA (p < 0.05).

**Table 2:** Result of seven parameters were measured (Mean ± SD)

	Right	Left	Bilateral
LN horizontal distance^*^ (mm)	5.64 ± 1.54	6.09 ± 1.78	5.86 ± 1.67
LN vertical distance^†^ (mm)	3.66 ± 1.7	3.27 ± 1.66	3.47 ± 1.57
MP horizontal distance^*^ (mm)	0.71 ± 1.73	0.77 ± 1.94	0.74 ± 1.83
SA^†^ (°)	88.75 ± 8.58	92.09 ± 8.38	90.42 ± 8.61
LA^*^ (°)	30.68 ± 6.16	30.73 ± 6.86	30.70 ± 6.49
PSD^*^ (mm)	6.51 ± 0.75	6.51 ± 0.78	6.51 ± 0.76
PSL^*^ (mm)	31.94 ± 4.58	31.22 ± 4.22	31.58 ± 4.40

*LN* lateral notch, *MP* the middle point of the inferior edge of the C6 articular process, *SA* sagittal angle, *LA* lateral angle, *PSD* pedicle screw diameter, *PSL* pedicle screw length

*p > 0.05. ^†^p < 0.05

**Fig. 3:**
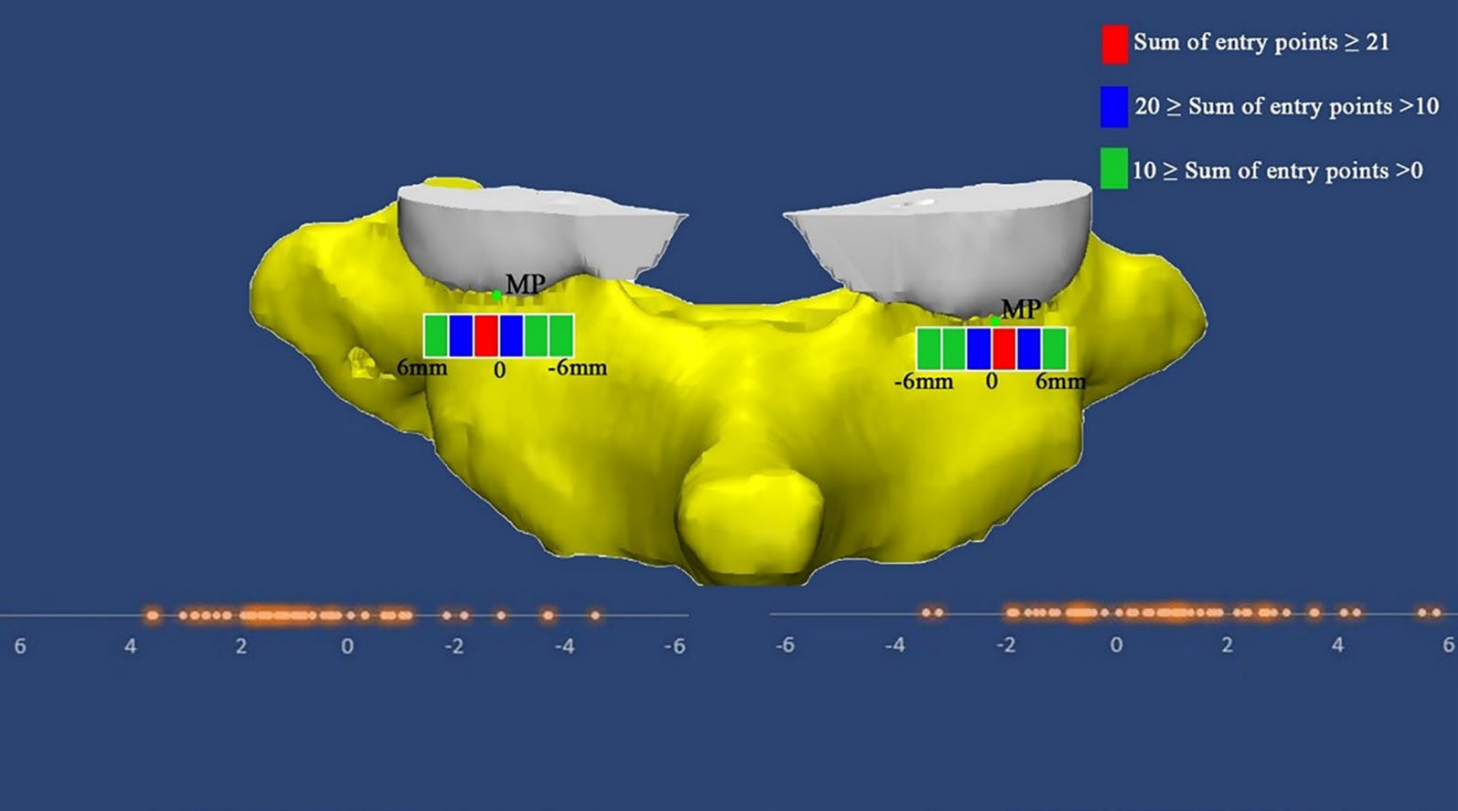
The distance between the entry point and MP (green points) is illustrated. The 12 mm area around the MP was divided into six parts: 47% of the entry points (total 57, left 33, right 24) were located within 0–2 mm lateral to the MP (red bar), 25% of the entry points (total 30, left 11, right 19) were located within 0–2 mm medial to the MP (blue bar), 18.3% of the entry points (total 22, left 11, right 11) were located within 2–4 mm lateral to the MP (blue bar), and only 9.2% of the entry points were located in the remaining area (green bar)

The average entry point for C7 was 5.86 ± 1.67 mm medial to the LN and 3.47 ± 1.57 mm superior to the LN (Table 2). The results of a comparison of the location between the two sexes are summarized in Table 3. The LN horizontal distance was significantly different between sexes (p < 0.05). With MP as an anatomic landmark, 83 (69.2%) entry points were located on the lateral side, and 37 (30.8%) were located on the medial side. The 12 mm area around the MP was divided into six parts, and most of the entry points (47.5%) were located within 2 mm lateral to the MP. Next, 25% of the entry points were located within 2 mm medial to the MP, and 18.3% of the entry points were located within 2–4 mm lateral to the MP. Only 9.2% of the entry points were located in the remaining 50% of the area. The locations of the entry points with respect to the MP are shown in Fig. 3.

**Table 3:** Comparison of seven parameters between different gender (Mean ± SD)

	Male	Female
LN horizontal distance^†^ (mm)	6.45 ± 1.58	5.23 ± 1.54
LN vertical distance^*^ (mm)	3.31 ± 1.52	3.63 ± 1.62
MP horizontal distance^*^ (mm)	0.76 ± 1.80	0.72 ± 1.88
SA^†^ (°)	92.47 ± 7.79	88.24 ± 8.97
LA^*^ (°)	30.10 ± 6.06	31.35 ± 6.92
PSD^†^ (mm)	6.81 ± 0.77	6.20 ± 0.62
PSL^†^ (mm)	32.38 ± 4.41	30.72 ± 4.27

*LN* lateral notch, *MP* the middle point of the inferior edge of the C6 articular process, *SA* sagittal angle, *LA* lateral angle, *PSD* pedicle screw diameter, *PSL* pedicle screw length

^*^p > 0.05. ^†^p < 0.05

The mean SA was 90.42 ± 8.61°, and the mean LA was 30.70 ± 6.49° (Table 2), which meant that the screws were inserted nearly vertical with respect to the FP in the sagittal plane. The results of the comparison of the SA and LA between the sexes are summarized in Table 3. The SA differed significantly between sexes (p < 0.05).

The average PSD was 6.51 ± 0.76 mm, and the average PSL was 31.58 ± 4.40 mm (Table 2). The maximum PSD was 8.60 mm, and the minimum PSD was 4.60 mm. The parameters of the PS for the males and females are summarized in Table 3. The PSD and PSL of the men were larger than those of the women (p < 0.05).

## Discussion

Cervical transpedicular screw insertion has been preferred to other posterior cervical fixation procedures for many years. Previous studies have investigated several methods to improve the accuracy of PS, including the use of intraoperative navigation systems, bony landmarks and intraoperative electromyography [1–3, 5–7, 10, 11, 13–18]. However, the risk of screw misplacement cannot be fully eliminated. Furthermore, the ideal entry point and trajectory have not yet been identified [7, 9, 19]. In this study, we developed a novel method to calculate the ideal entry point and trajectory and then analyzed related parameters to facilitate C7 PS insertion. We found that the ideal entry point for C7 was 5.86 ± 1.67 mm medial to the LN and 3.47 ± 1.57 mm superior to the LN, and 69.2% of the entry points were located lateral to the MP. The present study also found that the average PSD and PSL of the C7 PS were 6.51 ± 0.76 mm and 31.58 ± 4.40 mm, respectively, and the mean SA and LA for PS placement were 90.42 ± 8.61° and 30.70 ± 6.49°, respectively.

The ideal entry point and trajectory need to be identified to insert a cervical PS safely and accurately. Several methods have been proposed to calculate the ideal entry point and trajectory [2, 10–14]. Karaikovic et al. [10] studied 53 normal human cadaver cervical spines and defined the C7 pedicle entry point as the central point of the posterior projection of the pedicle isthmus on the lateral mass of the cervical vertebra. Rao et al. [12] quantitatively investigated the ideal PS entry points in terms of lateral mass geometry and found that the ideal insertion point and orientation are individually at most subaxial cervical levels. Lee et al. [11] tried to determine the optimal entry points and trajectories for cervical PS insertion using 3D spine models from cervical spine axial CT images and found that the optimal entry point for the C7 PS was 1.6 mm lateral to and 2.5 mm superior to the center of the lateral mass. The inconsistency in these results may be due to the sample size being relatively small or differences in the measurement methods. To date, no consensus regarding the best entry point and trajectory for C7 PS placement has been established. In the present study, the optimal entry points and trajectories for C7 PS insertion were thoroughly analyzed based on 3D spine models, and a unified coordinate system was established to determine the true parameters for C7 PS placement. We believe that the results of the study can help surgeons precisely perform C7 PS placement because of the strengths of the study.

Various factors affected the pullout strength of pedicle screw have been reported [20–25]. A biomechanical study conducted by Kueny et al. [23] showed that increasing the screw diameter by 1 mm can increase 24% pullout force. In another study, the researchers proved that the pullout strength of pedicle screw can be increased when screw contact with more cortical bone [25]. Besides, the bone mineral density of entry point and vertebral body can also affect the axial pullout strength [24].

**Fig. 4:**
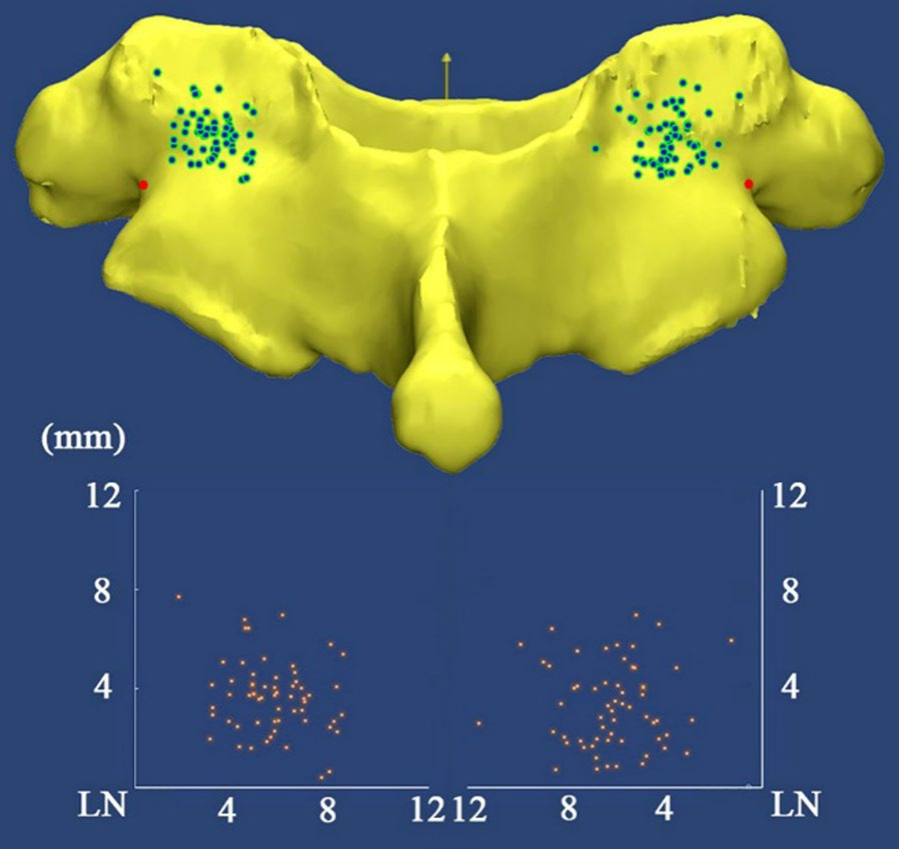
All entry points (green points) were located medial and superior to the LN (red points). Scatter diagram shows the exact positions of the entry points with respect to the LN. The horizontal axis indicates the lateral notch horizontal distance, and the vertical axis indicates the lateral notch vertical distance

Studies in the literature have recommended the use of several bony landmarks for cervical PS placement. Karaikovic et al. [10] first introduced the LN as a reference and proposed that 74.5% of the entry points were located 3 to 5 mm medial to the notch and 98.1% of the entry points were located at or above the notch. In a 3D imaging study, Lee et al. [11] reported that the entry point of C7 is 4.5 mm medial and 2.3 mm inferior to the LN. In another study, the authors investigated CT images and concluded that the C7 entry point was located 5.3 mm medial to the lateral border of the inferior articular process and 1.9 mm below the inferior border of the superior articular facet [12]. In our study, we found that all of the entry points were located medial and superior to the LN. The average distances were 5.86 mm and 3.47 mm, respectively (Fig. 4). These findings are different from those of Lee’s study but are similar to those reported by Karaikovic et al. [10], who demonstrated that only two (1.9%) entry points were located below the notch. Another landmark that we used for C7 PS placement was the MP, which is easy to identify intraoperatively. When we used the MP as a landmark, eighty-three (69.2%) entry points were located laterally, and 72.5% (47.5% were lateral and 25% were medial) of the entry points were located within 2 mm from the MP (Fig. 3).

Regarding the dimensions of the C7 pedicle, Rao et al. [12] measured 98 cervical CT images and showed that the mean pedicle width, pedicle height, and pedicle axis length were 7.2 mm, 6.7 mm, and 31.3 mm, respectively. Barrey et al. [26] studied 18 C7 vertebrae and found that the average pedicle width and height were 6.0 ± 1.2 mm and 5.8 ± 1.1 mm, respectively. These findings meant that all the C7 pedicles were suitable for a 3.5 mm diameter screw. Similar results were observed in our study. In our study, the mean PSD and PSL were 6.51 ± 0.76 mm (range 4.60–8.60 mm) and 31.58 ± 4.40 mm (range 19.86–43.74 mm), respectively, and with no exceptions, sex-related differences were also observed.

Previous studies have suggested that the screw trajectory should be placed parallel to the superior or inferior endplate in the sagittal plane when using freehand technique to insert PS during operation [3, 11, 12]. In this technique, an intraoperative fluoroscopic imaging is always obtained to ensure appropriate sagittal trajectory for the pilot hole. In our study, we designed a fitting plane of the lateral mass surface to guide the pedicle axis in the sagittal direction. The results showed that the mean SA was 90.42 ± 8.61°, which meant that whether the neck was placed in flexion or extension intraoperatively, the sagittal direction of the PS was nearly vertical to the lateral mass surface. To our knowledge, the present study is the first to introduce this method for surgeons to determine the sagittal direction when placing a PS in C7. In recent years, Yukawa et al. [7, 27] introduced a new “pedicle axis view” to guide screw trajectory. When performing pedicle screw insertion, the C-arm fluoroscopy was turned to show the configure of the pedicle as a circle—the “pedicle axis view’’. The process is technique challenge and time-consuming. A funnel technique was also described in the literature. However, these methods have not gained widespread practice. Another angular parameter used for PS insertion is the transverse angle. We found that the mean LA was 30.70 ± 6.49°, which was similar to previous findings [3, 28, 29].

One of the limitations of this study was that the measurement of 3D models might be affected by reconstruction techniques. Inaccurate measurements may exist. Although a high level of repeatability was demonstrated for the majority of the parameters measured, the repeatability of the MP horizontal distance was moderate. Second, we excluded subjects who previously underwent spine surgery and had neoplastic, congenital spine anomalies or severe degenerative changes; thus, we cannot simply generalize our findings to these patient populations. Third, we did not standardize the patients’ neck position when they underwent the CT scans; therefore, we did not measure the vertical distance between the entry point and MP. However, we believe that this study provides valuable information for PS implantation in C7.

## Conclusion

Based on CT-based 3D C7 digital models, we designed a new method to identify the ideal trajectory and entry point. Those results of the study may be helpful for the preoperative planning of C7 PS fixation for the patients who have relative normal anatomy features. Because there is significant variation in previously reported entry points, 3D CT imaging should be used preoperatively to carefully evaluate the anatomy of each individual.

## Data Availability

The datasets used and/or analysed during the current study are available from the corresponding author on reasonable request.

## Abbreviations

C7: The seventh cervical vertebra
PS: Pedicle screws
3D: Three dimensions
CT: Computed tomography
LN: Lateral notch
MP: The middle point of the inferior edge of the C6 articular process
PSD: Pedicle screw diameter
PSL: Pedicle screw length
LA: Lateral angle
SA: Sagittal angle
LNHD: LN horizontal distance
LNVD: LN vertical distance
ICC: The intraclass correlation coefficient
